# Genotypes of *Enterocytozoon bieneusi* in Livestock in China: High Prevalence and Zoonotic Potential

**DOI:** 10.1371/journal.pone.0097623

**Published:** 2014-05-20

**Authors:** Wei Li, Yijing Li, Weizhi Li, Jinping Yang, Mingxin Song, Ruinan Diao, Honglin Jia, Yixin Lu, Jun Zheng, Xichen Zhang, Lihua Xiao

**Affiliations:** 1 State Key Laboratory of Veterinary Biotechnology, Harbin Veterinary Research Institute, Chinese Academy of Agricultural Sciences, Harbin, Heilongjiang, China; 2 College of Veterinary Medicine, Northeast Agricultural University, Harbin, Heilongjiang, China; 3 Key Laboratory of Zoonosis Research, Ministry of Education, College of Veterinary Medicine, Jilin University, Changchun, Jilin, China; 4 Division of Foodborne, Waterborne and Environmental Diseases, Centers for Disease Control and Prevention, Atlanta, Georgia, United States of America; Technion-Israel Institute of Technology Haifa 32000 Israel, Israel

## Abstract

Despite many recent advances in genotype characterization of *Enterocytozoon bieneusi* worldwide and the exploration of the extent of cross-species transmission of microsporidiosis between humans and animals, the epidemiology of this neglected disease in China is poorly understood. In this study, a very high prevalence (60.3%; 94/156) of *E. bieneusi* infections in farmed pigs in Jilin province was detected by PCR of the ribosomal internal transcribed spacer (ITS). DNA sequence analysis of 88 *E. bieneusi*–positive specimens identified 12 distinct genotypes (11 known: CHN7, CS-1, CS-4, CS-6, EbpA, EbpB, EbpC, EbpD, EBITS3, G, and Henan-I; one novel: CS-9). Frequent appearance of mixed genotype infections was seen in the study animals. Weaned (74.6%; 53/71) or pre-weaned (68.8%; 22/32) pigs have infection rates significantly higher than growing pigs (35.8%; 19/53) (p<0.01). Likewise, *E. bieneusi* was detected in 2 of 45 sheep fecal specimens (4.4%) in Heilongjiang province, belonging to the known genotype BEB6. Genotypes EbpA, EbpC, EbpD, and Henan-I examined herein have been documented in the cases of human infections and BEB6, EbpA, EbpC, and EbpD in wastewater in central China. Infections of EbpA and EbpC in humans were also reported in other areas of the world. The other known genotypes (CHN7, CS-1, CS-4, CS-6, EBITS3, EbpB, and G) and the new genotype CS-9 were genetically clustered into a group of existing *E. bieneusi* genotypes with zoonotic potential. Thus, pigs could be a potential source of human *E. bieneusi* infections in China.

## Introduction

Microsporidia are a large and diverse group of unicellular eukaryotes that infect a wide range of invertebrate and vertebrate taxa [1]–[3]. At least 14 microsporidian species in 8 genera have been recognized as human pathogens, with *Enterocytozoon bieneusi* being the most prevalent [1]–[3]. *E. bieneusi* is distributed worldwide and has a wide host range, infecting many species of humans, domestic and wild animals, and even birds and causing diarrhea [1]–[3]. It is an opportunistic organism in AIDS patients and has been frequently detected in healthy individuals [4]–[7]. Microsporidiosis caused by *E. bieneusi* is mainly transmitted through fecal-oral routes [1]. The sources of infection by *E. bieneusi* are usually other infected humans and animals and contaminated food and water [1], [2]. Understanding epidemiology and the roles of livestock in disease transmission may be helpful in developing strategy for the prevention and control of human microsporidiosis [8].

Genotyping of *E. bieneusi* in humans and animals is mainly based on the polymorphisms of the internal transcribed spacer (ITS) of the rRNA gene, having identified at least 150 genotypes thus far [1], [9]. A large cluster of genetically linked *E. bieneusi* genotypes (Group 1) are frequently found in both humans and animals, thus are considered to have zoonotic potential [10]. In contrast, the remaining genotypes represent largely host-adapted groups (Groups 2 to 5) associated with specific animals and probably have no significant public health importance [10]. Pigs are infected with over 30 *E. bieneusi* genotypes, some of which (CAF1, D, EbpA, EbpC, EbpD, H, O, PigEBITS5, and PigEBITS7) are known human pathogens [1], [7], [11], [12]. One of 7 goats in Galicia, northern Spain was examined to be *E. bieneusi*-positive [13], whereas no report concerning the identification of this organism in sheep has been documented thus far.

In central China, the occurrence and genotype distribution of *E. bieneusi* in humans have been examined, but the source of infections is still not clear [6], [7]. The present study analyzed 156 fecal specimens from healthy pigs of 3 age groups (<30 days, 30 to 60 days, and >60 days) in city Changchun, Jilin Province and 45 fecal specimens from sheep of 3 age groups (1 to 3 months, 3 to 6 months, and >6 months) in cities Suihua and Daqing, Heilongjiang Province for *E. bieneusi* genotypes, and evaluated the potential roles of livestock in zoonotic transmission of microsporidiosis.

## Materials and Methods

### Ethics statement

This study was performed in accordance with the recommendations in the Guide for the Care and Use of Laboratory Animals of the Ministry of Health, China. Prior to experiment, the protocol of the current study was reviewed and approved by the Institutional Animal Care and Use Committee of the Harbin Veterinary Research Institute and Northeast Agricultural University, under the approved protocol number SRM-08. Before beginning work on the study, we contacted the farm owners and obtained their permission. No specific permits were required for the described field studies. And the locations where we sampled are not privately-owned or protected in any way. The field studies did not involve endangered or protected species.

### Specimen collection

A total of 156 specimens were collected during October to December 2012 from pigs on 2 farms in suburban Changchun and assigned to 3 age groups: 32 from pre-weaned pigs (<30 days), 71 from weaned pigs (30 to 60 days), and 53 from growing pigs (>60 days). Sheep specimens collected in Suihua included 10 from pre-weaned lambs (1 to 3 months) and 30 from post-weaned lambs (15 females and 15 males: 3 to 6 months). An additional 5 specimens from adult sheep aged >6 months were collected from Daqing during April to May 2012. Fresh fecal specimen (approximately 30 g) of each animal was collected immediately after being defecated on the ground of the pen using a sterile disposal latex glove, and then placed in a clean 50 ml plastic container individually. All the farm livestock animals were healthy at the time of sampling. All fecal specimens were stored at 4°C in 2.5% (w/v) potassium dichromate. Only one specimen per animal was used in this study.

### DNA extraction and PCR amplification

Prior to DNA extraction, fecal specimens were washed twice with distilled water. Genomic DNAs were extracted from 0.2 g of washed fecal specimens using a Stool DNA Rapid Extraction Kit (Spin-column) (TIANGEN, China) and manufacturer-recommended procedures. *E. bieneusi* was detected by nested PCR amplification of a 392-bp fragment that covered the entire ITS region of the rRNA gene [14]. Each specimen was analyzed twice using 2 µl of the DNA extract per PCR. Non-acetylated bovine serum albumin (TaKaRa, Japan) was added in all primary PCRs at the concentration of 400 ng/ µl to neutralize residual PCR inhibitors in the extracted DNA. PCR products were electrophoresed on 1.0% agarose gels and visualized with ethidium bromide staining.

### Sequence analysis and phylogeny

The secondary PCR products of the expected size were sequenced in both directions in the Sangon Company (Shanghai, China). After being edited using Chromas Pro 1.33 (Technelysium Pty Ltd, Helensvale, Queensland, Australia), the nucleotide sequences were aligned with reference sequences using the ClustalX 1.81 package (ftp://ftp-igbmc.u-strasbg.fr/pub/ClustalX/) to determine *E. bieneusi* genotypes. A neighbor-joining tree was constructed to assess the relationship of *E. bieneusi* genotypes identified in the present study and those described in previous studies, using the software Mega 4 (http://www.megasoftware.net/) and the evolutionary distances calculated by Kimura 2-parameter model. The ITS tree was rooted with GenBank sequence DQ885585. The reliability of cluster formation was evaluated by the bootstrap method with 1,000 replicates.

### Nucleotide sequence accession numbers

Nucleotide sequence of the ITS of the new *E. bieneusi* genotype CS-9 was deposited in the GenBank database under accession number KF724904.

### Statistical analysis

Infection rates between animal groups were compared using Chi-square test using SPSS 17.0 version (SPSS Inc., Chicago, IL, USA). The difference was considered significant when the p-value was <0.05.

## Results

### Frequency of *E. bieneusi* in pigs and sheep

Among the 156 pig specimens, 94 (60.3%) were positive for *E. bieneusi* by PCR amplification of the ITS locus. Twenty-two of 32 pre-weaned, 53 of 71 weaned, and 19 of 53 growing pigs were infected with the pathogen. The difference in infection rates between weaned (74.6%) and growing animals was significant (35.8%) (p<0.01, χ^2^ = 18.8). Pre-weaned animals (68.8%) also had a significantly higher infection than growing animals (p<0.01, χ^2^ = 8.7).

The pathogen was detected in pre-weaned lambs (2/10; 20%) from Suihua. Nevertheless, we did not detect *E. bieneusi* in the other two age groups (post-weaned and adult sheep).

### Distribution of *E. bieneusi* genotypes in livestock

Nucleotide sequences of the ITS were obtained from 88 of 94 *E. bieneusi*-positive pig specimens, detecting 12 distinct genotypes (11 known: CHN7, CS-1, CS-4, CS-6, EbpA, EbpB, EbpC, EbpD, EBITS3, G, and Henan-I; one new: CS-9) (Table 1). The occurrence of mixed genotype infections was seen in 10 animals, with 1 pre-weaned pig infected with genotypes CS-1 and G, 1 pre-weaned pig with CS-6 and EbpA, 1 weaned pig with CS-9 and EbpD, 1 weaned pig with EbpA and EbpC, and 1 pre-weaned, 2 weaned, and 3 growing pigs with CS-9 and EbpB (Table 1). Only several single nucleotide polymorphisms (SNPs) in the ITS sequences contributed to the existence of high diversity of *E. bieneusi* genotypes in pigs (data not shown). The most prevalent genotype found in pigs was EbpA (38.6%), followed by EbpC (34.1%), CS-9 (9.1%), CHN7 (8.0%), EbpB (6.8%), CS-4 (4.5%), Henan-I (3.4%), CS-1 (2.3%), CS-6 (1.1%), EBITS3 (1.1%), EbpD (1.1%), and G (1.1%) (Table 1).

**Table 1:** *Enterocytozoon bieneusi* genotypes identified in farmed pigs and sheep in northeast China.

Animal	City (province)^a^	Genotype	Positive no. (age group^c^)	Host (location^d^)	Reference
Pig	CC (JL)	CS-9	1 *G*	Pig	This study
		CS-1	1 *W*	Pig (China)	KF607047^e^
		CS-4	2 *P* and 2 *W*	Pig (China)	KF607050^e^
		CHN7	1 *P* and 6 *W*	Pig (China)	[16]
		EBITS3	1 *G*	Pig (USA, Korea, and Switzerland)	[1]
		EbpA	4 *P*, 21 *W*, and 7 *G*	Human (China, Nigeria and CZE), Pig (Japan, Germany, CZE, Switzerland, and USA), Bird (CZE), Cattle (Germany), Horse (CZE), and Mice (CZE), and Wild boar (CZE and Poland)	[1], [7], [11], [12], [25]–[29]
		EbpC	10 *P*, 13 *W*, and 6 *G*	Human (China, Vietnam, Thailand, and CZE), Pig (Thailand, Japan, Germany, and Switzerland), and Wild mammals (China, Austria, CZE, Poland, and USA)	[1], [6], [7], [24], [29], [30]
		Henan-I	1 *P*, 1 *W*, and 1 *G*	Human (China)	[6]
		CS-1/G^b^	1 *P*	See above/Pig (Germany) and Horse (CZE)	[1], [25]
		CS-6/EbpA^b^	1 *P*	Pig (China)/See above	KF607052^e^
		CS-9/EbpB^b^	1 *P*, 2 *W*, and 3 *G*	See above/Pig (Switzerland)	[31]
		CS-9/EbpD^b^	1 *W*	See above/Human (China) and Pig (Switzerland)	[6], [31]
		EbpA/EbpC^b^	1 *W*	See above	[1], [6], [7], [11], [12], [24]–[30]
Sheep	SH (HL)	BEB6	2 *L*	Cattle (USA)	[1]

a CC: Changchun; SH: Suihua; JL: Jilin; HL: Heilongjiang.

b Mixed infection.

c *P*: pre-weaned pigs aged <30 days; *W*: weaned pigs ≈30 to 60 days; *G*: growing pigs >60 days; *L*: lambs ≈30 to 90 days.

d Location where the genotypes were identified before this work; CZE: Czech Republic.

e GenBank accession number.

The *E. bieneusi* found in 2 pre-weaned lamb specimens belonged to a known genotype: BEB6, which was previously examined in cattle in the US [15].

### Phylogenetic analysis

**Figure 1:**
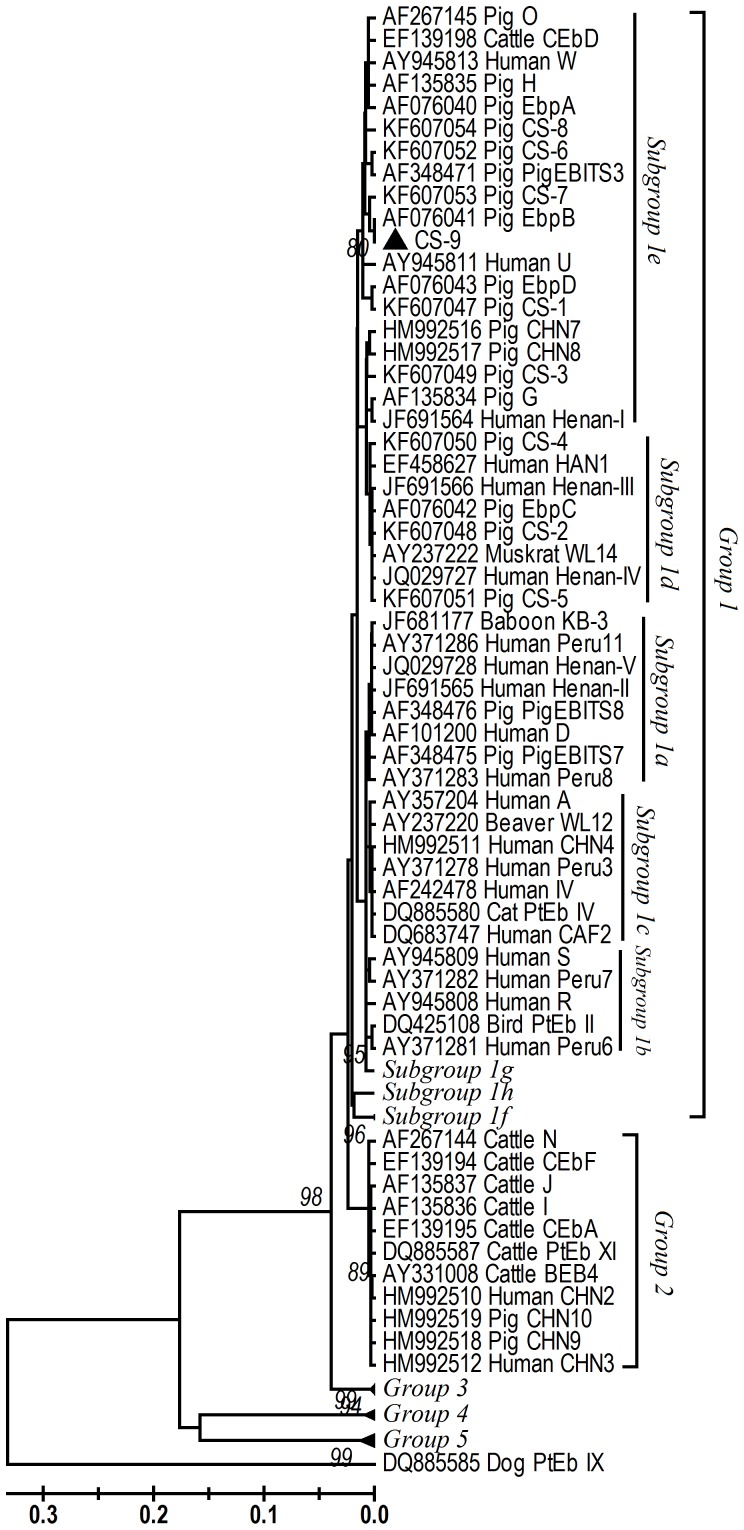
Phylogenetic relationship of ITS sequences of *Enterocytozoon bieneusi* in this study and known *E. bieneusi* genotypes, as inferred by a neighbor-joining analysis (Mega 4 software [http://www.megasoftware.net/]) based on Kimura two-parameter genetic distances. The ITS tree was rooted with GenBank sequence DQ885585. Bootstrap values less than 70% from 1,000 replicates are not shown. CS-9 indicated by triangle is a new genotype found in this study.

A neighbor-joining tree was constructed to assess the genetic relationship of the new genotype CS-9 and those described in previous studies. The sequence of genotype CS-9 was a member of a major phylogenetic group with zoonotic potential (Group 1 reported by Thellier and Breton, 2008) [10] (Fig. 1). The major cluster consisted of the genotype CS-9 from the present study, genotypes CHN7, CHN8, and CS-1 to CS-8 in pigs in northeast China [16], genotypes D, EbpC, EbpD, IV, Peru 8, Peru 11, PigEBITS7, and Henan-I to Henan-V in humans in Henan [6], genotypes EbpA and EbpC in humans in Shanghai [7], genotype CHN4 in humans in Changchun in Jilin [16], and some other genotypes reported previously in humans and wild and domestic animals [1] (Fig. 1). As observed previously, genotype BEB6 found in sheep belonged to the host-adapted Group 2 [10].

## Discussion

In this study, all the livestock sampled had frequent contact with their keepers. Comparing to the infection rate of 16.4% in growing pigs in a livestock production facility in urban Changchun [16], this study presented an even higher positive rate of the pathogen (60.3%) in farmed pigs without diarrhea in suburban Changchun. The prevalence difference between this and the study in urban Changchun [16] probably has resulted from differences in the age of study animals, detection methods, seasonality, and ecological environments. We also found that infection rates of *E. bieneusi* between weaned and growing, or between pre-weaned and growing healthy pigs were significantly different. Although *E. bieneusi* is prevalent in humans and livestock (cattle, pigs, dogs, cats, horses, etc) worldwide and even goats in Spain, no studies have described the identification of this organism in sheep [1], [13]. Our study presented the first report on *E. bieneusi* in 2 pre-weaned lambs in northeast China. Livestock are in close contact with humans in China, calling special attention to the potential occurrence of zoonotic transmission of microsporidiosis.

Genotyping based on the ITS locus has contributed to the improved understanding of *E. bieneusi* transmission in broad geographic areas and a range of hosts [1], [2], [5], [17]. Although the epidemiology of microsporidiosis in China remains unclear, the limited data on *E. bieneusi* genotypes in domestic animals, humans, and wastewater have been helpful to elucidate the sources and transmission routes of this neglected disease [6], [7], [16], [18]. Here we investigated genotypic diversity of *E. bieneusi* in pigs and sheep in northeast China. A total of 13 distinct genotypes were found, with EbpC, EbpD, and Henan-I previously identified in HIV+ and HIV- humans in Henan [6], EbpA and EbpC in hospitalized children in Shanghai [7], CS-1, CS-4, CS-6, and CHN7 in pigs in northeast China [16], and BEB6, EbpA, EbpC, and EbpD in wastewater in 4 cities, central China [18]. Genotypes EbpA and EbpC have also represented the causes for human microsporidiosis in other geographical regions of the world. For example, genotype EbpA we reported in pigs was previously found in humans in Nigeria and Czech Republic [11], [12]. Genotype EbpC identified in this study also infected humans in Vietnam, Thailand, Peru, and Czech Republic [19]–[24]. In addition to the human-pathogenic genotypes EbpA, EbpC, EbpD, and Henan-I we identified, genotypes CHN7, CS-1, CS-4, CS-6, EBITS3, EbpB, and G and the new genotype CS-9 are members of Group 1, thus have zoonotic potential.

Although the source of *E. bieneusi* infections in humans in China has not been determined, a high prevalence of genotype EbpC in humans in Henan was strongly associated with raising pigs in the household [6]. This genotype was also identified to be highly prevalent in pigs in this study. The frequent occurrence of genotype EbpC in humans in central China [6], [7] and in pigs in northeast China supports the likely occurrence of zoonotic transmission. Actions should be taken to reduce close contact between *E. bieneusi*-harboring pigs and susceptible human populations to reduce the spread of microsporidiosis.

In conclusion, we reported the occurrence of several human-pathogenic *E. bieneusi* genotypes and a new genotype with zoonotic potential in healthy pigs in northeast China. Although the prevalence of *E. bieneusi* in livestock in China has been poorly investigated thus far, our finding suggests that pigs could be a potential source of human microsporidian infections. Further studies are needed to fully elucidate the significance of pigs in the epidemiology of microsporidiosis in humans. Due to the high frequency of human contact with livestock in China, advice should be given to the susceptible human populations to reduce zoonotic transmission of this neglected disease.
